# Innovative Delivery Systems for Curcumin: Exploring Nanosized and Conventional Formulations

**DOI:** 10.3390/pharmaceutics16050637

**Published:** 2024-05-09

**Authors:** Jibira Yakubu, Amit V. Pandey

**Affiliations:** 1 Pediatric Endocrinology, Diabetology and Metabolism, University Children’s Hospital, Inselspital, 3010 Bern, Switzerland; jibira.yakubu@unibe.ch; 2 Translational Hormone Research Program, Department of Biomedical Research, University of Bern, 3010 Bern, Switzerland; 3 Graduate School for Cellular and Biomedical Sciences, University of Bern, 3012 Bern, Switzerland

**Keywords:** curcumin, curcuminoids, nanoparticles, nanomedicine, nanoencapsulation, nanodelivery

## Abstract

Curcumin, a polyphenol with a rich history spanning two centuries, has emerged as a promising therapeutic agent targeting multiple signaling pathways and exhibiting cellular-level activities that contribute to its diverse health benefits. Extensive preclinical and clinical studies have demonstrated its ability to enhance the therapeutic potential of various bioactive compounds. While its reported therapeutic advantages are manifold, predominantly attributed to its antioxidant and anti-inflammatory properties, its efficacy is hindered by poor bioavailability stemming from inadequate absorption, rapid metabolism, and elimination. To address this challenge, nanodelivery systems have emerged as a promising approach, offering enhanced solubility, biocompatibility, and therapeutic effects for curcumin. We have analyzed the knowledge on curcumin nanoencapsulation and its synergistic effects with other compounds, extracted from electronic databases. We discuss the pharmacokinetic profile of curcumin, current advancements in nanoencapsulation techniques, and the combined effects of curcumin with other agents across various disorders. By unifying existing knowledge, this analysis intends to provide insights into the potential of nanoencapsulation technologies to overcome constraints associated with curcumin treatments, emphasizing the importance of combinatorial approaches in improving therapeutic efficacy. Finally, this compilation of study data aims to inform and inspire future research into encapsulating drugs with poor pharmacokinetic characteristics and investigating innovative drug combinations to improve bioavailability and therapeutic outcomes.

## 1. Introduction

**Figure 1:**
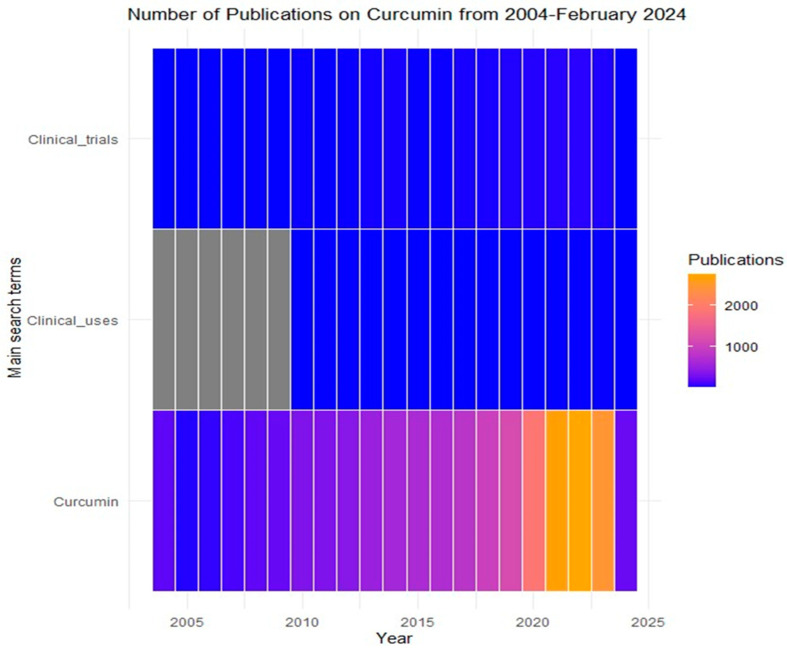
Number of publications related to Curcumin per year. The data were obtained from PubMed using the following keywords: Curcumin, curcuminoids, nanoparticle, clinical uses, clinical trials. The blue color indicates a low number of published articles, and the yellow color indicates a high number of published articles captured in the PubMed database/website (https://pubmed.ncbi.nlm.nih.gov/). Accessed on 28 February 2024.

Turmeric, a traditional spice derived from the rhizomes of *Curcuma longa*, has garnered significant attention in scientific research owing to its rich phytochemical composition and diverse therapeutic potential. Over millennia, turmeric has been utilized across various cultures for its purported health benefits, supported by an extensive body of research spanning multiple organ systems in humans [1,2,3,4]. The bioactive compounds within turmeric, particularly curcumin, have been extensively studied for their antimicrobial, antioxidant, and anti-inflammatory properties, which underpin their therapeutic efficacy [5]. Moreover, computational docking and in vitro experiments conducted previously in our laboratory revealed that curcumin exhibits a strong binding affinity towards the active sites of steroid metabolizing cytochrome P450 proteins [6]. Specifically, our findings demonstrated inhibition of androgen metabolizing CYP17A1 and estrogen metabolizing CYP19A1 enzymes by curcuminoids, suggesting a promising avenue for the development of novel compounds with enhanced efficacy and safety profiles for targeting both prostate and breast cancers and other hyperandrogenic disorders [6]. The exponential growth in scientific publications related to curcumin shows its overall significance in biomedical research, with over 2500 publications as of February 2024, highlighting its clinical potential (Figure 1).

Curcumin, the principal curcuminoid found in turmeric, has captivated researchers since its isolation over two centuries ago, originating from India. Structurally, curcumin exists as a yellowish diarylheptanoid crystalline powder, soluble in certain organic solvents but exhibiting limited solubility in others [7]. Curcuminoids, including curcumin, demethoxycurcumin, and bisdemethoxycurcumin, constitute the primary derivatives isolated from turmeric, each contributing to its therapeutic profile [2,4,8,9,10]. Despite the promising bioactivities of curcumin and other phytochemicals, their translation into effective therapeutic agents faces challenges stemming from inherent limitations, including poor solubility, structural instability, and low bioavailability [11,12,13,14,15]. These constraints hinder their clinical utility, necessitating innovative strategies to enhance their pharmacokinetic and pharmacodynamic properties. Nanoformulation technologies have emerged as a promising approach to address these challenges, offering avenues to improve the solubility, stability, and targeted delivery of curcumin.

**Figure 2:**
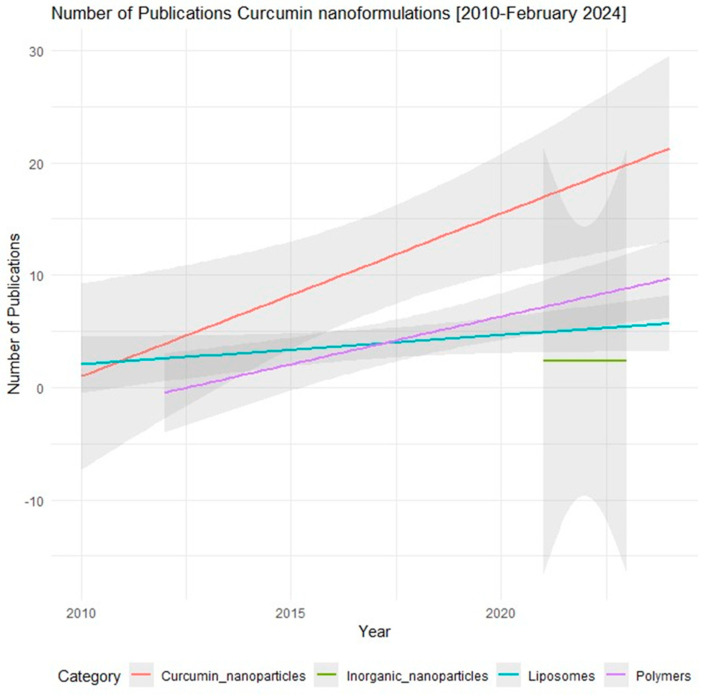
Number of publications related to Curcumin nanoformulation from 2010 to February 2024. The data were obtained from PubMed (https://pubmed.ncbi.nlm.nih.gov/) using the following keywords: Curcumin plus nanoparticle, Inorganic nanoparticles, polymers/polymersomes, lipid−based nanoparticle/liposomes. Accessed on 28 February 2024.

Nanoformulations encompass a diverse array of carriers, including polymeric complexes, biofriendly inorganic substances, and lipids, each offering unique advantages for drug delivery [16]. Notably, nanocarriers such as dendrimers, nanocrystals, polymersomes, and liposomes have gained prominence in biomedical research and pharmaceutical applications due to their ability to traverse biological barriers and exert therapeutic effects within the body [17,18,19,20,21,22]. Polymer-based nanoparticles and lipid-based nanocarriers have dominated the landscape of nanoformulations, comprising nearly 99% of reported data, indicative of their widespread adoption and research interest (Figure 2). Polymer-based nanoparticles and lipid-based nanocarriers represent versatile platforms for drug delivery, offering distinct advantages in terms of biocompatibility, tenable properties, and targeted delivery. Polymer-based nanoparticles, such as poly (lactic-co-glycolic acid) (PLGA) and polyethylene glycol (PEG) derivatives, offer a customizable framework for encapsulating curcumin, thereby improving its solubility and stability while facilitating controlled release kinetics [23]. These nanoparticles can be tailored to modulate drug release profiles, enhance cellular uptake, and achieve prolonged circulation times in vivo, thus optimizing the therapeutic efficacy of curcumin [5,24].

Similarly, lipid-based nanocarriers, including liposomes, solid lipid nanoparticles (SLNs), and nanoemulsions, exhibit inherent biocompatibility and the ability to encapsulate lipophilic compounds like curcumin within their hydrophobic cores [25]. Liposomes, composed of phospholipid bilayers, can encapsulate curcumin within aqueous compartments or lipid bilayers, enabling targeted delivery to specific tissues or cells while minimizing off-target effects [25]. SLNs offer advantages in terms of stability and sustained release, making them suitable candidates for encapsulating curcumin and overcoming its inherent bioavailability limitations [26]. Nanoemulsions, comprising oil-in-water or water-in-oil formulations, provide a stable platform for delivering hydrophobic compounds like curcumin, enhancing its bioavailability and therapeutic efficacy [27,28].

The dominance of polymer-based nanoparticles and lipid-based nanocarriers in nanoformulation research shows their versatility and effectiveness in addressing the challenges associated with curcumin delivery. By harnessing the unique properties of these nanocarriers, researchers can overcome barriers to clinical usage of curcumin, unlocking its full therapeutic potential for various applications, including antimicrobial, antioxidant, anti-inflammatory, neuroprotective, and anticancer interventions [29,30]. Furthermore, advancements in nanotechnology offer opportunities to innovate novel delivery systems, such as hybrid nanoparticles and stimuli-responsive carriers, further enhancing the bioavailability and efficacy of curcumin-based therapeutics [31,32].

Despite the numerous advantages they offer, nanoparticles come with several inherent disadvantages, varying across different types. Polymer nanoparticles, while offering promising drug delivery capabilities, are often plagued by issues such as poor stability, low encapsulation efficiency for hydrophilic drugs, and challenges associated with scaling up the production [33]. Additionally, the use of organic solvents in conventional preparation methods can pose toxicity risks to humans and may lead to degradation of encapsulated active agents [33,34]. Liposomal nanoparticles, although effective in enhancing drug delivery and bioavailability, face challenges such as fast elimination in vivo, potential sterility issues, and the risk of drug leakage [35,36,37,38,39]. Moreover, there is a concern that liposomal nanomedicines may inadvertently target healthy tissues, leading to cytotoxicity and immune reactions [38]. Metal nanoparticles, despite their unique properties and applications in various fields, can raise concerns regarding toxicity and biocompatibility. There is growing evidence suggesting that certain metal nanoparticles may induce toxicological effects in biological systems, necessitating comprehensive research to understand their impact on both external and internal environments [40,41]. Moreover, the release of active oxygen by some metal nanoparticle systems can contribute to oxidative stress and inflammation, further emphasizing the need for cautious evaluation of their safety profiles [40]. Nanoemulsions also present drawbacks related to sterility, drug leakage, and the limited understanding of their toxicity profiles, necessitating further investigation to elucidate their impact on biological systems [42]. Ongoing research efforts are geared toward optimizing performance and ensuring the safe and effective utilization of nanoparticulate systems in clinical settings.

Curcumin is generally considered safe when consumed in moderate amounts; however, high doses may lead to gastrointestinal discomfort and, in rare cases, allergic reactions [2]. Additionally, some studies suggest potential toxicity at very high doses or with long-term use [43]. For instance, animal studies have reported adverse effects such as liver toxicity at doses higher than 1000 mg/kg body weight per day over extended periods. It is also worth noting that curcumin can interact with certain medications and may interfere with blood clotting.

In the following sections we review recent biomedical applications of curcumin nanoformulations as a targeted drug delivery system to improve the bioavailability and efficacy of curcumin. This review particularly focuses on curcumin nanoformulation strategies to resolve the inherent biochemical and biophysical limitations of curcumin and its derivatives. In this review, we delve into the intricate landscape of curcumin pharmacokinetics, nanoformulations, and synergistic combinations, shedding light on recent advancements and promising approaches. We begin by elucidating the pharmacokinetic profile of curcumin, unravelling the complexities of its absorption, distribution, metabolism, and excretion in vivo. Drawing insights from preclinical and clinical studies, we examine the factors influencing the bioavailability of curcumin and propose strategies to overcome these barriers.

Furthermore, we explore the burgeoning field of nanoformulations tailored to encapsulate curcumin, offering enhanced solubility, stability, and targeted delivery. From lipid-based nanoparticles to polymeric micelles and solid lipid nanoparticles, we dissect the diverse array of nanoformulation approaches employed to harness the therapeutic potential of curcumin. Through a critical analysis of preclinical and clinical data, we evaluate the efficacy and safety of these nanoformulations, delineating key parameters governing their design and optimization.

Moreover, we investigate the synergy between curcumin and other bioactive compounds, elucidating how combinatorial approaches can potentiate the therapeutic effects of curcumin while mitigating potential adverse effects. From phytochemicals and nutraceuticals to conventional drugs and natural products, we scrutinize the multifaceted interactions underlying synergistic combinations with curcumin, offering mechanistic insights and therapeutic implications. Additionally, we discuss how combining curcumin with other therapeutic agents enhances its absorption and therapeutic potential, with a focus on targeted and sustained release formulations.

In summary, this review provides a comprehensive overview of the pharmacokinetics, nanoformulations, and synergistic combinations with curcumin, highlighting their potential to revolutionize therapeutic interventions across a spectrum of diseases. By elucidating the underlying mechanisms and translational challenges, we aim to inspire future research endeavors and therapeutic innovations, ultimately advancing the clinical utility of curcumin-based therapies. Moreover, we address the regulatory considerations and translational hurdles in bringing curcumin nanoformulations and synergistic combinations from bench to bedside, emphasizing the importance of interdisciplinary collaborations and translational research efforts in realizing the full therapeutic potential of curcumin.

## 2. Pharmacokinetic Profile of Curcumin

**Figure 3:**
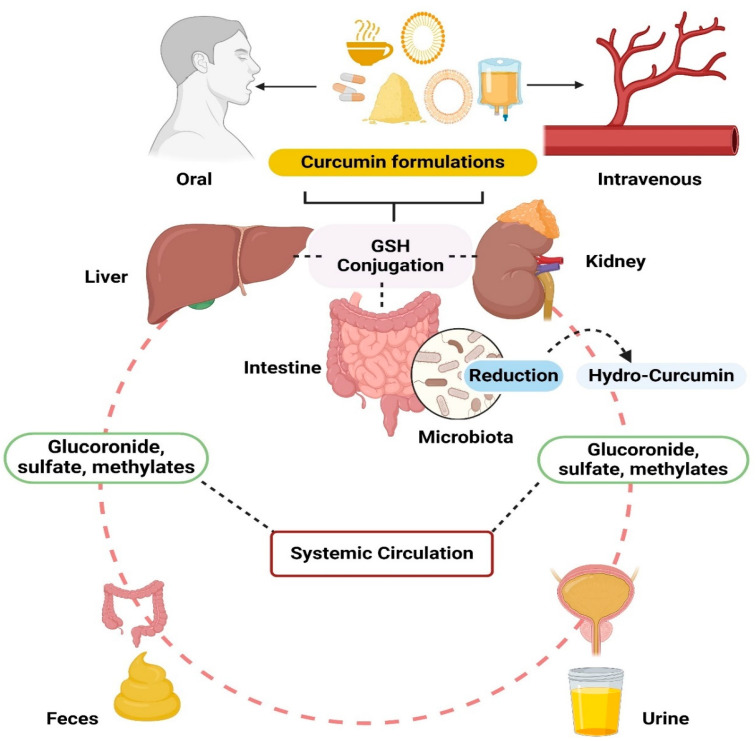
Oral or intravenous administration of different formulations of curcumin result mainly in conjugated or reduced curcumin detected in systemic circulation (plasma), and intravenous administration results mainly in only reduced curcumin metabolites (hydrocurcumins).

Curcumin demonstrates rapid solubility in organic solvents such as acetone, ethanol, dimethyl sulfoxide, and dimethylformamide. However, the stability of curcumin varies depending on diluent pH. While curcumin remains stable under acidic pH conditions, it degrades into ferulic acid and feruloylmethane in neutral and basic pH environments [44]. The stability of curcumin is compromised in buffer solutions at neutral pH, although it remains stable in the presence of ascorbic acid, N-acetylcysteine, and glutathione. The gastrointestinal absorption of curcumin is notably poor, as evidenced by minimal absorption observed in human subjects and animal models following oral administration. However, several studies have shown improved absorption rates, with more than 20% absorption observed at oral doses of 100–1500 mg [15,45,46]. When taken orally, curcumin goes through quick changes in the small intestine, liver, and kidneys, transforming into curcumin glucuronide, curcumin sulphate, and methylated curcumins [15,45,47,48]. These altered forms are then rapidly eliminated from the body through urine and feces. The scheme of curcumin conjugation and reduction in the humans are depicted in Figure 3. In the bloodstream, curcumin mainly exists as these modified compounds, which are not as biologically active, resembling the behavior of other polyphenols [15,44,45,48].

Additionally, intestinal microorganisms play a role in extensively reducing curcumin to dihydrocurcumin, tetrahydrocurcumin, and hexahydrocurcumin [15,44,45,47,48]. The maximum recommended oral dose for humans is 8 g/day for up to 3 months, with no reported toxic or hazardous effects at this dosage level [49]. Recent advancements in curcumin research have shed light on its potential therapeutic applications beyond its traditional uses. From neuroprotective effects to modulation of gut microbiota and metabolic pathways, curcumin’s versatility continues to be explored in various disease contexts. Moreover, novel delivery systems such as nanoformulations aim to improve its bioavailability and therapeutic efficacy, paving the way for its translation into clinical practice.

Numerous studies have elucidated the enhanced pharmacokinetic profile of curcumin when administered as nanoformulations or colloidal dispersions [50,51,52,53,54,55,56,57]. Curcumin nanoformulations have emerged as a promising strategy to address these challenges and enhance the therapeutic efficacy of curcumin. Nanocarriers offer several advantages, including increased solubility, protection from degradation, sustained release, and improved cellular uptake [58,59,60,61]. Generally, nanoformulation alters the physicochemical properties of curcumin, facilitating its transport across biological barriers and enhancing its bioavailability. One of the key mechanisms by which nanoformulation enhances curcumin bioavailability is through improved solubility. Curcumin’s poor aqueous solubility limits its dissolution and absorption in the gastrointestinal tract, leading to low systemic levels and suboptimal therapeutic outcomes [31]. Nanoformulation circumvents this limitation by dispersing curcumin molecules within the aqueous core or lipid bilayers of nanoparticles, thereby increasing their dispersibility and dissolution rate. In liposomal formulations, curcumin is encapsulated within the aqueous core or incorporated into the lipid bilayers [62,63,64], and can fuse with cellular membranes, facilitating the direct uptake of curcumin by cells [65,66]. Also, surface modifications of liposomes with ligands or targeting moieties further improve their specificity and efficacy in delivering curcumin to specific tissues or cells [36].

In addition, colloidal carriers are composed of biocompatible and biodegradable polymers, such as poly (lactic-co-glycolic acid), chitosan, and albumin. These nanoparticles encapsulate curcumin within their matrix or adsorb it onto their surface [67]. Polymeric nanoparticles can be functionalized with targeting ligands or stimuli-responsive polymers to enhance the site-specific delivery and minimize off-target effects [68]. Furthermore, SLN encapsulation of curcumin, protects it from degradation and improve its solubility [69]. SLNs offer advantages such as controlled release, high drug loading capacity, and enhanced cellular uptake. These nanoparticles can traverse biological barriers, such as the blood–brain barrier, and deliver curcumin to target tissues or organs. Furthermore, SLNs exhibit excellent biocompatibility and stability, making them suitable carriers for long-term drug delivery applications [70,71].

Moreover, nanoformulation protects curcumin from premature degradation and metabolism, thereby prolonging its circulation time and enhancing its tissue distribution [63,72,73,74]. Nanoparticles serve as carriers that shield curcumin from enzymatic degradation and facilitate its passive or active transport across biological membranes [58,59,60,61]. Nanoformulation allows for controlled release kinetics, enabling sustained and localized delivery of curcumin to target tissues or cells [60,61].

Nanoparticles exhibit favorable size, surface charge, and surface modifications that promote interactions with cellular membranes and facilitate endocytosis [75]. Once internalized, nanoparticles release curcumin within the intracellular compartments, allowing for direct interaction with molecular targets and signaling pathways [63].

Overall, the comparison of various nanoformulations of curcumin reveals distinct advantages and limitations. Curcumin liposomes offer improved solubility and stability, with potential for targeted delivery, despite challenges in production consistency and storage stability. Conversely, curcumin polymeric nanoparticles offer controlled release and biocompatibility but may suffer from issues in drug loading efficiency and burst release. Solid nanoparticles provide high drug loading capacity, prolonged circulation time, and enhanced cellular uptake, yet face challenges in the scaling up of production and control of particle size distribution. The selection of the most suitable nanoformulation depends on specific therapeutic requirements, emphasizing the need for careful consideration.

## 3. Current Curcumin Nanoformulations and Their Pharmacological Profile

**Figure 4:**
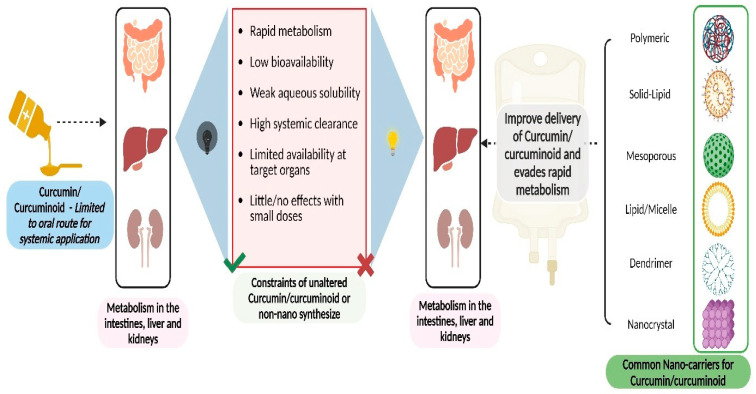
Schematic representation of the limitations of curcumin bioavailability and nanocarrier-mediated improvements in delivery. Raw curcumin/curcuminoids face numerous challenges hindering their therapeutic efficacy, including rapid metabolism, low bioavailability, weak aqueous solubility, high clearance, limited availability to target organs, and minimal effects at small doses. Nanocarriers, such as polymeric, solid lipid, mesoporous, liposomes, dendrimer nanoparticles, and nanocrystals, encapsulated curcumin/curcuminoids, shield it from rapid metabolism and enhance its stability in circulation. The utilization of nanocarriers represents a promising strategy to enhance the delivery of curcumin, circumventing its inherent limitations and maximizing its therapeutic potential in various disease treatments.

Nanoformulations of curcumin have garnered significant interest in recent years as a promising strategy to address its inherent challenges of poor solubility and bioavailability. Among these nanoformulations, lipid-based nanoparticles, solid lipid nanoparticles, nanoemulsions, polymeric nanoparticles, dendrimers, and nanocrystals have emerged as frontrunners due to their unique properties and biocompatibility (Figure 4).

For instance, a phase I open-label study evaluated the safety and efficacy of liposomal curcumin in patients with metastatic tumors. This investigation identified 300 mg/m^2^ of liposomal curcumin as the tolerated dose and observed promising tumor marker responses in select patients [76]. The curcumin liposomes sustained curcumin plasma concentrations, highlighting its potential as a delivery system for targeted cancer therapy [76]. In another double-blinded, placebo-controlled trial, Campbell et al. [77] administered curcumin formulated with fenugreek soluble fiber to obese men for 12 weeks. The enhanced bioavailability of curcumin resulted in favorable changes in cardiovascular biomarkers such as homocysteine and high-density lipoprotein concentrations, hinting potential cardiovascular health benefits. The combination of curcumin with fenugreek soluble fiber enhanced curcumin’s bioavailability, leading to improved metabolic parameters and lipid profile [77].

Furthermore, a randomized, double-blind, placebo-controlled trial investigated the effects of nanocurcumin supplementation on metabolic status in diabetes patients undergoing hemodialysis [78]. Nanocurcumin demonstrated significant improvements in glucose metabolism, lipid profile, and inflammatory markers compared to placebo. The nanomicelle formulation of curcumin enhanced its solubility and bioavailability, leading to better therapeutic outcomes in diabetic patients on hemodialysis [78]. In this randomized controlled trial (RCT) [78], patients with diabetes undergoing hemodialysis were assigned to receive either 80 mg/day of nanocurcumin capsules or placebo for 12 weeks. Nanocurcumin supplementation led to significant improvements in fasting plasma glucose, serum insulin levels, triglycerides, very low-density Lipoprotein-cholesterol, total cholesterol, low-density Lipoprotein-cholesterol, total-/high-density Lipoprotein-cholesterol ratio, high-sensitivity C-reactive protein, plasma malondialdehyde, total antioxidant capacity, and total nitrite levels compared to placebo. Additionally, nanocurcumin upregulated the gene expression of peroxisome proliferator-activated receptor gamma and the low-density Lipoprotein receptor in peripheral blood mononuclear cells but did not affect the gene expression of transforming growth factor beta. Curcumin encapsulated nanomiscelles are also reported to promote bone strengthening in postmenopausal women [79]. Kia and colleagues [79] in their RCT conducted at the cancer center of the Razi Hospital in Rasht, Iran, divided patients into two groups: those undergoing chemotherapy and head and neck radiotherapy, and those receiving chemotherapy only for cancer in other organs. Chemotherapy drugs included Cisplatin (30–50 mg) and 5-Fluorouracil (640–750 mg), while radiotherapy dosage ranged from 6000 to 7000 cGy. The study group received 80 mg of nanomicelle curcumin capsules twice daily after meals. The control group experienced significantly more severe oral mucositis (OM) than the study group at weeks 1 (*p* = 0.010), 4 (*p* = 0.022) and 7 (*p* < 0.001), with OM severity gradually increasing in the control group over 7 weeks. Erythema and ulceration scores also increased gradually in the control group but remained stable in the study group. Pain scores were significantly lower in the study group at week 7 (*p* = 0.001). Patients receiving only chemotherapy in the study group had significantly lower OM severity scores than the control group throughout the 7 weeks (*p* < 0.001), while those undergoing both chemotherapy and head and neck radiotherapy had significantly lower OM severity at weeks 4 (*p* = 0.009) and 7 (*p* = 0.012). The nanomicelle curcumin capsules showed effectiveness in preventing and treating chemotherapy and radiotherapy-induced OM, particularly in chemotherapy-induced cases. However, increasing the dosage beyond one capsule a day did not significantly impact OM severity. Furthermore, another group [80] investigated the effects of nanomicelle curcumin (80 mg) and *Nigella sativa* (NS) oil supplementation in postmenopausal women aged 50–65 with low baseline bone mineral density. Changes in alkaline phosphatase, osteocalcin (OC), and osteoprotegerin (OP) biomarkers were analyzed. Significant reductions in ALP levels were observed in the NS and nanomicelle curcumin with NS (CUR-NS) groups compared to placebo. After adjusting for baseline values and covariates, a significant reduction in ALP was observed only in the CUR-NS group compared to placebo and nanomicelle curcumin alone. OC levels decreased in all groups except CUR-NS, while OP levels decreased in all groups. Safety assessments showed insignificant differences in renal and hepatic biomarkers between groups. Participant satisfaction with medication was high across all groups. The study suggests the beneficial effects of CUR-NS on bone turnover biomarkers in postmenopausal women, though further investigation into bone strength is warranted. Both nanomicelle curcumin and NS oil appear safe for use in postmenopausal women, but additional studies are needed to confirm these findings and elucidate underlying mechanisms.

Pharmacokinetic studies of a standardized novel solid lipid curcumin particle, commercially available as Longvida^®^, showcased increased bioavailability compared with a generic curcumin extract, suggesting the potential for sustained release in both in vitro and in vivo studies [81,82,83]. Gota et al. [81] evaluated the plasma levels of free curcumin after administering a solid lipid curcumin particle (SLCP) formulation compared to unformulated curcumin in healthy volunteers and late-stage osteosarcoma patients, while assessing tolerability and dose-plasma concentration relationships. The SLCP formulation was administered orally in capsule form at dosages of 2000 mg, 3000 mg, and 4000 mg of curcumin, with water, over a maximum duration of 5 min. The calibration curve showed linearity over the concentration range of 2−500 ng/mL of curcumin in human plasma. The LOD and LLOQ were found to be 1 and 2 ng/mL of curcumin in human plasma, respectively. Stability studies indicated no significant loss of curcumin after plasma storage at room temperature for 4 h. Additionally, oral administration of SLCP showed appreciable plasma concentrations compared to unformulated curcumin. SLCP significantly improved the bioavailability of free curcumin, with no reported adverse events in either the healthy volunteers or the osteosarcoma patients. Further research is warranted to assess long-term tolerability, dose–response relationships, and the clinical impact of SLCP in larger sample sets across various health issues where curcumin has shown promise. The plasma concentration achieved with SLCP in this study aligns with levels shown to impact disease markers associated with Alzheimer’s disease and cancer, supporting the need for additional pharmacokinetic and clinical investigations using SLCP [84,85]. Moreover, clinical studies have demonstrated the safety of Longvida^®^ and evaluated the cognitive and mood-enhancing effects in healthy older adults. Participants taking Longvida^®^ supplement showed improvements in working memory and mood parameters compared to placebo. The SLCP used in the study demonstrated superior cognitive benefits and neuroprotective effects, highlighting its potential in age-related cognitive decline [86].

Ahmadi et al. [87] conducted a 12-month, double-blind, randomized, placebo-controlled trial evaluating nanocurcumin as an adjunctive treatment for amyotrophic lateral sclerosis (ALS). Nanocurcumin supplementation showed promising results, improving survival rates, and demonstrating a favorable safety profile in ALS patients. The nanocurcumin formulation exhibited potent anti-inflammatory and antioxidant properties, providing neuroprotective effects, and enhancing patient outcomes in ALS.

Additionally, nanocurcumin has shown strong antiviral effects, particularly in mitigating inflammatory responses and reducing mortality rates in COVID-19 patients. Studies investigating the immunomodulatory effects of nanocurcumin in COVID-19 patients reported reductions in inflammatory cytokine levels, suggesting its potential as an adjunctive therapy in COVID-19 management. The nanocurcumin formulation mitigated cytokine storm and inflammatory responses, offering a promising adjunctive therapy for COVID-19 patients [88,89,90,91,92]. Hassaniazad et al. [91], studied the effects of curcumin nanomicelles on clinical outcomes and immune response changes in COVID-19 patients through a randomized, triple-blinded, placebo-controlled trial. The nanocurcumin group received Sinacurcumin^®^ soft gel capsules (40 mg) four times daily for 2 weeks. Clinical signs and symptoms, along with laboratory indices, were evaluated at three time points: 0, 7, and 14 days, in both groups. While there were no statistically significant differences in clinical manifestations between the two groups, the C-reactive protein level decreased in the second week after nanocurcumin administration compared to the placebo group. Notably, the nanocurcumin group exhibited an increase in the mean percentage of lymphocytes and other favorable trends in the laboratory indices compared to the placebo group. No specific adverse reactions related to curcumin were observed. Serum levels of IFN-γ, IL-4, IL-17, and TGF-β did not significantly differ between the nanocurcumin and placebo groups, although trends have suggested modulation of immune responses by nanocurcumin, including decreased Th1 and Th17 responses and increased T regulatory responses. Ahmadi et al. [92] conducted a double-blind, randomized clinical trial to evaluate the effects of nanocurcumin on clinical manifestations in patients hospitalized with mild-to-moderate COVID-19. Nanocurcumin, formulated as biodegradable polymer nanoparticles, was administered at a dose of 40 mg four times daily for two weeks. They reported no significant differences between the two experimental groups in most clinical parameters (*p* > 0.05). However, supplementation with nanocurcumin led to a significant reduction in cough (*p* = 0.036), fatigue (*p* = 0.0001), myalgia (*p* = 0.027), and oxygen demand (*p* = 0.036), as well as improvements in blood oxygen saturation (*p* = 0.006).

Collectively, these studies show the potential of nanoformulations of curcumin in overcoming the challenges associated with its poor bioavailability and unlocking its full therapeutic potential. From cancer treatment to cardiovascular health, metabolic disorders, neurodegenerative diseases, and viral infections like COVID-19, nanocurcumin holds promise as a versatile and effective therapeutic agent. Further research and clinical trials are warranted to elucidate its mechanisms of action, optimize dose regimens, and validate its efficacy across diverse medical conditions. With continued advancements in nanotechnology, nanocurcumin stands poised to revolutionize modern medicine and improve healthcare outcomes worldwide.

## 4. Other Novel Formulations of Curcumin

In addition to nanoformulations, other investigations have elucidated a range of innovative curcumin formulations tailored to enhance its bioavailability and therapeutic efficacy in addressing diverse health conditions.

**Galactomannan Biopolymer Formulation:** Matthewman et al. [15] discussed the use of natural fiber, specifically galactomannan biopolymer from *Trigonella foenum graecum* (fenugreek), to enhance the pharmacokinetics and efficacy of curcuminoids. This patented formulation, known as Curcumin-galactomannoside (CGM), combines 35–40% curcuminoids with 60% fenugreek galactomannan dietary fiber [93]. Preclinical and clinical studies with CGM have demonstrated superior efficacy compared to standard unformulated curcumin, attributed to improved bioavailability and tissue distribution [94,95,96]. Mechanistic evidence suggests CGM interacts with various cellular targets, regulating genes involved in cancer pathogenesis [15,94,95].

**Phytosomal Curcumin:** Phytosomes are plant metabolites with complex chemistry. Phytosomes contain amphipathic molecules, making them resistant to degradation and highly absorbed in the gut, thus improving their bioavailability [97,98,99]. Curserin^®^ is a commercially available phytosomal curcumin containing phosphatidylserine, phosphatidylcholine, and piperine. Cicero et al. [100] investigated the effects Curserin^®^ on subjects with overweight and impaired fasting glucose. After 8 weeks of treatment, significant improvements were observed in various metabolic parameters, including fasting plasma insulin, lipid profile, liver function tests, and serum cortisol levels, compared to baseline and placebo.

**Colloidal Submicron Particles and amorphous Formulation (Theracurmin^®^ and CurcuRouge^TM^):** Theracurmin^®^, a commercial bioavailable curcumin, and has garnered attention for its enhanced bioavailability and therapeutic potential. Theracurmin^®^ is water-dispersible, with significantly improved absorption and tissue penetration capabilities, represents a novel approach to alleviating challenges associated with curcumin therapeutics. Reports that compared the absorption efficiency of Theracurmin^®^ with other curcumin drug delivery system (DDS) preparations, showed that Theracurmin^®^ exhibited significantly higher absorption efficiency, with more than 2–5-fold higher plasma curcumin concentration and 2–6 fold higher area under the concentration–time curve compared to the other DDS [53,54,101,102]. A recent study by Sunagawa et al. [50] compared the bioavailability of an amorphous formulation (curcuRouge^TM^) with Theracurmin^®^. Both animal and human studies showed that curcuRouge^TM^ exhibited superior bioavailability, achieving 3.7-fold higher plasma concentration in rats and 3.4-fold higher bioavailability in human volunteers compared to Theracurmin^®^. But curcuRouge^TM^ has not been investigated for its efficacy in various disorders. Specifically, Theracurmin^®^ has shown promise in treating various conditions such as muscle damage, inflammation, and alcohol intoxication. Studies have indicated its efficacy in managing knee osteoarthritis, with patients experiencing reduced knee pain and decreased reliance on anti-inflammatory medications when administered Theracurmin^®^ [56]. Moreover, Theracurmin^®^ has demonstrated an inhibitory effect on alcohol intoxication in human subjects. This effect is evidenced by a reduction in blood acetaldehyde concentration following alcohol consumption [53]. The multifaceted therapeutic potential of Theracurmin^®^, includes its effects in musculoskeletal disorders [55] and improving symptoms of neurodegenerative disorders [103].

## 5. Combinations of Curcumin with Other Therapeutic Agents

Combination therapy, integrating multiple druggable agents, has emerged as a fundamental strategy in disease therapeutics. By combining drugs targeting similar or different pathways, it enhances pharmacodynamic outcomes, potentially reducing drug resistance. Curcumin, an FDA-approved nutraceutical, offers extensive health benefits but faces limitations in mainstream healthcare due to costly modifications. Hence, approaches combining FDA-approved drugs with nutraceuticals are gaining traction across various diseases [104,105,106]. Different scientific reports have shed light on the pharmacokinetic and pharmacodynamic profile of curcumin combinatory therapy (Table 1).

During the 1990s, preclinical studies revealed compelling findings regarding the administration of curcumin alongside piperine. In murine models, this combination led to a remarkable 154% increase in serum concentration. Even more impressively, human trials demonstrated a staggering 2000% surge in serum concentration, with no reported adverse effects [107].

Researchers propose the combination of curcumin and piperine as a potential therapeutic approach for managing COVID-19, leveraging their multifaceted mechanisms of action encompassing antiviral, anti-inflammatory, immunomodulatory, antifibrotic, and antioxidant effects [108,109,110,111]. For instance, Pawar et al. [109] conducted a double-blind, randomized, controlled trial at a dedicated COVID health center (DCHC) in Maharashtra, India, to investigate the effects of curcumin co-administered with piperine, aimed at optimizing curcumin absorption, on COVID-19 symptoms. Patients in the study group received curcumin (525 mg) with piperine (2.5 mg) in tablet form twice daily, in addition to conventional COVID-19 treatment, while those in the control group received probiotics twice daily. The study findings demonstrated that adjunctive therapy with orally administered curcumin and piperine significantly mitigated morbidity and mortality, reduced treatment expenses, and alleviated the logistical burden on healthcare systems. Furthermore, dose-escalation studies demonstrated the safety of curcumin over a period of three months, highlighting its potential as a safe and natural therapeutic option for preventing post-COVID thromboembolic events. These findings are consistent with the outcomes of two clinical studies conducted by Askari and colleagues [111].

Furthermore, another research group investigated the impact of curcumin–piperine combination therapy on patients recovering from ischemic stroke. The results were promising, with a significant increase in total antioxidant capacity (*p* < 0.001). Moreover, the combination therapy led to reductions in serum levels of high-sensitivity C-reactive protein (*p* = 0.026), total cholesterol (*p* = 0.009), triglycerides (*p* = 0.001), carotid intima-media thickness (*p* = 0.002), weight (*p* = 0.001), waist circumference (*p* = 0.024), as well as systolic and diastolic blood pressure (*p* < 0.001) [112].

Other studies highlight the pharmacokinetic and pharmacodynamic profiles of curcumin combinatory therapy. For instance, phototherapeutic effect of curcumin has been explored in combination with photodynamic therapy, demonstrating broad-spectrum efficacy against oral microorganisms [113,114]. Additionally, curcumin, combined with various agents, shows promising outcomes across different disease contexts.

**Oral Health:** Curcumin, combined with blue light and other compounds, effectively disinfects the oral cavity, offering potential applications in dental care [115,116,117]. Niu et al. [113] showed in their report that curcumin with blue- and red-light irradiation has a promising strategy for inhibiting cell proliferation and inducing apoptosis in Ha-CaT cells. In their study, curcumin at low concentrations significantly (*p* < 0.05) inhibited cell proliferation, and induced apoptosis, notably at late stages, while arresting the cell cycle at the G2/M transition point. Mechanistically, the combination therapy inhibited NF-κB activation induced by TNF-α, enhanced activation of caspase-9 and caspase-8, and attenuated TNF-α-induced activation of ERK and Akt. Another study explored the photodynamic effect of curcumin on inducing apoptosis in human malignant glioblastoma cells via NF-κB and Nrf2 axes. The test revealed that 10 μM of curcumin treated T98G cells on exposure to blue light, resulting in increased cell death with longer irradiation durations, particularly at 5 and 10 min. Curcumin combined with photodynamic therapy in-duces ROS generation and T98G cell death through NF-κB- and Nrf2-mediated MMP2 and MMP9 pathways in glioblastoma cancers [114]. Clinical studies conducted by Araujo et al. [115] also explored the effectiveness of photodynamic therapy (PDT) against oral bacteria sourced from human saliva, following sensitization with curcumin and exposure to blue light at 450 nm. Their findings revealed that curcumin, at a concentration of 30 mg/L from a 1500 mg/L stock solution, led to a noteworthy reduction (>65%) in saliva microorganisms, akin to the efficacy of conventional oral solutions such as chlorhexidine and alcohol rinses. These results corroborate similar clinical investigations conducted by independent research groups [116,117]. Additionally, the use of curcumin in combination with other agents has demonstrated efficacy in the treatment of oral submucous fibrosis, contributing to symptom reduction and improved treatment outcomes [118,119]. Nerkar Rajbhoj et al. [118] investigated the efficacy of curcumin gel combined with *Aloe vera* gel alongside oral physiotherapy for managing oral submucous fibrosis (OSMF). Their findings revealed that Aloe Vera gel led to a more pronounced improvement in burning sensation scores compared to curcumin gel, possibly due to differences in their anti-inflammatory potency. Additionally, Adhikari et al. [119] demonstrated the efficacy of curcumin in combination with intralesional dexamethasone and hyaluronidase for treating OSMF, reporting significant improvements in interincisal mouth opening, cheek flexibility, and tongue protrusion. The anti-inflammatory and antioxidant properties of curcumin contribute to oral health improvement. However, larger-scale studies with longer durations are warranted to validate these findings across all stages of OSMF. Concerns regarding the poor absorption of curcumin, resulting in low systemic availability, have been raised, suggesting the potential of curcumin–phospholipid complexes to enhance bioavailability. Furthermore, inconsistencies in systemic curcumin concentrations among different formulations underscore the need for further research on optimal formulations and dosages.

**Neurological Disorders:** Additionally, curcumin exhibits neuroprotective effects and may have potential in managing neurodegenerative disorders. In a recent study by Seko et al. [24], PLGA nanoparticles (NPs) were utilized for the co-delivery of docetaxel and curcumin, showing dose-dependent toxicity consistent with blank PLGA NPs but minimal at concentrations conducive to effective drug transport. Combining docetaxel with curcumin (CCM) demonstrated reduced toxicity compared to docetaxel alone, further mitigated by docetaxel loaded PLGA NPs. Curcumin’s ability to enhance docetaxel efficacy while reducing dosage and side effects suggests its potential in overcoming chemoresistance mechanisms. Their report shows the promise of polysorbate 80-coated docetaxel and curcumin-loaded PLGA NPs as a viable strategy for brain glioma therapy, offering enhanced drug delivery and therapeutic synergy [24].

**Gastrointestinal Disorders:** Curcumin combined with standard medications significantly reduces chronic inflammation in patients with gastritis caused by *H. pylori*, while boosting antioxidant defenses [120]. Judaki et al. [120] conducted a randomized controlled trial (RCT) to evaluate the effects of curcumin on oxidative stress and histological changes in chronic gastritis associated with *H. pylori* infection. In their study, triple therapy consisting of omeprazole, amoxicillin, and metronidazole was administered alongside curcumin supplementation for four weeks. The findings demonstrated that curcumin exhibited antioxidant and antibacterial effects, resulting in a higher eradication rate of *H. pylori* infection and alleviation of gastric mucosal damage, suggesting its potential as a safe and effective treatment option for patients on triple therapy. Furthermore, Gasbarrini et al. [121] investigated the efficacy and tolerability of a combination of curcumin and fennel essential oil (CU-FEO) in relieving symptoms of irritable bowel syndrome (IBS). Their study enrolled IBS patients who received either CU-FEO or placebo capsules twice daily for 30 days. The results showed a significant reduction in IBS symptom severity scores with CU-FEO compared to placebo, along with improvements in abdominal pain severity, distension, dissatisfaction with bowel habits, and quality of life (QoL). Notably, CU-FEO treatment led to a higher rate of complete symptom resolution and demonstrated a significant improvement in various domains of the IBS-QoL questionnaire [121].

**Metabolic Syndromes:** Combining curcumin with bioactive agents such as chlorogenic acid, coconut yogurt, ferrous sulphate, and boswellic acid has shown promise in mitigating metabolic dysfunctions [122,123,124,125]. For example, curcumin supplementation alongside omega-3 polyunsaturated fatty acids has demonstrated beneficial effects on triglyceride levels, insulin sensitivity, and components of metabolic syndrome [126,127]. Chilleli et al. [122] investigated the effects of curcumin (10 mg) and *Boswellia serrata* gum resin (105 mg of boswellic acids) supplementation on oxidative stress and inflammation markers in master cyclists, finding significant reductions in glycoxidation and lipid peroxidation markers after three months of supplementation. In another study by Larinczova et al. [123] a bioavailable form of curcumin (HydroCurc^TM^) did not inhibit ferrous iron absorption in healthy individuals, indicating its safety in combination with iron supplements. Furthermore, co-administration of formulated curcumin with ferrous sulphate did not diminish the physiological effects of serum iron levels. Specifically, the lower dose curcumin groups, and the corresponding curcumin placebo group, after 180 min, showed an increase in serum iron levels of 11.41 µmol/L and 8.79 µmol/L, respectively. The higher dose curcumin groups and the corresponding iron and placebo group exhibited an increase in serum iron levels of 16.39 µmol/L and 19.09 µmol/L, respectively. Additionally, they observed a significant increase in hemoglobin levels in the placebo group, which was retained in the curcumin group, suggesting that the addition of formulated curcumin did not diminish the physiological effects of serum iron levels. Additionally, a similar study investigated whether co-administration of ferrous sulfate with a formulated, bioavailable form of curcumin (HydroCurc™) could mitigate systemic inflammation and gastrointestinal side effects [125]. Their findings suggest that supplementing ferrous sulfate with a formulated, bioavailable curcumin may effectively reduce systemic inflammation and oxidative stress compared to using iron alone. This approach may enhance the benefits of ferrous sulfate supplementation while alleviating some of its associated adverse effects. Consequently, these results hold significant implications, paving the way for novel approaches to oral iron supplementation. Further research, particularly in a larger cohort of iron-deficient individuals, is warranted to validate and expand upon these findings. In a randomized crossover study, healthy postmenopausal women who consumed bioactive yogurt containing curcumin exhibited significantly lower plasma TNFα levels compared to those who consumed placebo yogurt, indicating an anti-inflammatory effect [124].

**Cancer Treatment:** Curcumin’s anticancer properties offer potential adjuvant therapy in various cancer types. Combinations of curcumin with chemotherapy agents or other bioactive compounds enhance cancer treatment efficacy, promoting apoptosis, inhibiting tumor growth, and improving treatment outcomes [24,128,129]. Jame et al. [128] conducted a study using patient-derived colorectal liver metastases to investigate whether curcumin could enhance the effects of 5-fluorouracil (5-FU) and oxaliplatin (FOLFOX) in cancer stem cell models. Subsequently, they clinically evaluated the combination of curcumin with FOLFOX chemotherapy in a phase I dose escalation study. Participants were administered 500 mg (1 capsule) of oral curcumin C3-complex daily for 7 days before scheduled chemotherapy. If no curcumin-induced toxicities (CITs) were observed, daily oral curcumin was continued upon commencement of chemotherapy. The FOLFOX-based chemotherapy regimen consisted of 2-weekly cycles, with a maximum of 12 cycles or until withdrawal from the trial. After observing no CITs in the initial group of patients following completion of two chemotherapy cycles, recruitment for the next dose escalation to 1 g (2 capsules) daily, and then 2 g (4 capsules) daily, began. The study concluded that Curcumin + FOLFOX inhibited the growth of primary cancer stem cell (CSC) spheroid models, decreased expression of CSC markers in primary CSC spheroid models, enhanced the proapoptotic effects of chemotherapy in explant culture, and was safe and tolerable when combined with FOLFOX chemotherapy. Their findings are consistent with a study by Howells et al. [129].

Despite promising effects observed with certain chemotherapeutics, some studies have reported no discernible benefits when curcumin is combined with therapeutic agents. Passildas-Jahanmohan and colleagues [130] investigated whether supplementation of 6 g/d of curcumin with docetaxel for 7 days, every 3 weeks, could improve prognosis among metastatic castration-resistant prostate cancer (mCRPC) patients. The results of this phase II study led to the conclusion that in mCRPC patients, oral curcumin combined with docetaxel at the studied dosage (6 g/d for 7 days) did not improve either progression-free survival or overall survival. The small sample size may have contributed to the absence of a difference between the two treatment arms. Consequently, curcumin cannot be recommended in association with chemotherapy in mCRPC based on this study. However, further research involving larger cohorts is necessary to explore whether curcumin could be beneficial in other cancer treatments, and to investigate if improved formulations of curcumin could enhance its efficacy. Curcumin formulations that combine other agents to enhance bioavailability should be of particular interest in future trials that combine curcumin with chemotherapeutic agents. Many potential applications of curcumin-based compounds in cancer therapy have been pursued and are currently under consideration worldwide.

**Osteoarthritis Management:** The strategic combination of curcumin with other agents has emerged as a promising approach in managing osteoarthritis, with its down-regulatory effects on key mediators implicated in the pathogenesis of the condition [131,132]. Additionally, the anti-inflammatory properties of curcumin offer further therapeutic benefits by alleviating joint pain and enhancing mobility [133]. The study conducted by Lammi, and colleagues [131] aimed to identify new targets of curcuminoid extracts, hydrolyzed collagen, and green tea extract (COT) using genomic and proteomic approaches. Differential analysis revealed significant differential expression of genes between IL-1β and control conditions, COT IL-1β and IL-1β conditions, and COT and control conditions, with 2549, 2280, and 1907 genes, respectively. Further analysis highlighted genes associated with inflammation, cartilage metabolism, and angiogenesis pathways. Notably, inflammatory mediators upregulated in IL-1β conditions were downregulated in COT IL-1β conditions, with CXCL6 showing a remarkable multi-fold change. Immunoassay validation confirmed decreased production of CXCL6 protein in COT-treated conditions compared to IL-1β. The results of their research suggest that the identified genes are associated with critical pathophysiologic processes in osteoarthritis and represent potential targets for osteoarthritis treatment. In another investigation [132], the combined effects of curcumin and alendronate on bone turnover markers and bone densitometric features related to osteoporosis in postmenopausal women were examined. Alendronate was administered at doses of 5 mg/day and 110 mg/day for 12 months, along with calcium supplements (1000–1500 mg/day). Their results demonstrated the efficacy of curcumin in combination with alendronate in reducing bone turnover markers (BALP and sCTx) and increasing bone mineral density compared to the control group. Furthermore, a study compared the efficacy of extracts containing a combination of boswellic acid and curcumin (Curamin^®^) with curcumin alone (CuraMed^®^) or placebo in treating degenerative joint disease symptoms in patients aged 40 to 77 years. Their results highlight the effectiveness of curcumin and its combination with boswellic acid in reducing pain-related symptoms in osteoarthritis patients, with the combination therapy demonstrating superior efficacy in reducing inflammation and pain [133].

**Chronic Kidney Disease:** Murrillo Ortiz et al. [134] conducted a randomized, double-blind, placebo-controlled trial to investigate the impact of twelve weeks of supplementation with resveratrol and curcumin on the recovery of bone and muscle mass, as well as protein and lipid oxidation, in patients with chronic kidney disease undergoing hemodialysis with iron overload. The participants received a daily oral dose of 500 mg of resveratrol and 500 mg of curcumin throughout the 12-week period. Anthropometric parameters were assessed at the onset of treatment to evaluate its effects on muscle and bone mass percentages. Following supplementation, both muscle mass (46.01 ± 8.85 kg to 53.51 ± 9.81 kg, *p* = 0.01) and bone mass (2.46 ± 0.44 kg to 2.85 ± 0.48 kg, *p* = 0.01) exhibited significant increases, consequently elevating BMI levels. Their reports of combination therapy involving resveratrol and curcumin has shown to induce favorable effects on muscle and bone mass while reducing fat accumulation in patients undergoing hemodialysis for chronic kidney disease. This therapeutic approach addresses multiple complications commonly associated with chronic kidney disease.

**Inflammation Associated with Different Pathologies:** A randomized trial investigated the efficacy and tolerability of a combination therapy comprising curcuminoid complex and diclofenac versus diclofenac alone in patients with knee osteoarthritis. The results demonstrated that both treatment groups exhibited improvements in pain relief and quality of life. However, patients receiving the combination therapy displayed significantly superior enhancement in various outcome measures, particularly in pain and quality of life scores (*p* < 0.001) compared to the diclofenac monotherapy group. Furthermore, the combination therapy was well-tolerated, with fewer adverse effects reported [135].

In another study, the efficacy of a combination therapy involving curcumin and omega-3 fatty acids was investigated in individuals suffering from episodic migraine. The findings revealed that supplementation with this combination resulted in a significant reduction in serum vascular cell adhesion molecule (VCAM) levels, indicating a modulation of inflammatory processes associated with migraine pathology. Additionally, participants reported improvements in migraine symptoms and overall wellbeing [136].

**Table 1:** Summary of the drug combinations with curcumin for treating various diseases.

Form of Curcumin	Combinatory Agent (s)	Clinical Use	Outcome	Type of Study	References
Nano-Curcumin	Docetaxel	Glioma	Easy passage through the BBB and reduction in the toxic effects of high dose docetaxel	Preclinical	[24]
Curcumin	Omeprazole, amoxicillin, and metronidazole	Gastritis–associated *Helicobacter pylori* infection.	Eradication of Helicobacter pylori infection	Clinical	[120]
Curcumin	Long-chain omega-3 polyunsaturated fatty acids	Type II Diabetes	Reduces blood lipids, increases insulin sensitivity but has no effect on blood glucose	Clinical	[126,127]
Curcumin	Chlorogenic acid and coconut yogurt	Inflammation	Anti-inflammatory effects	Clinical	[124]
Curcumin	Fennel essential oil	Irritable bowel syndrome	Safe and effective	Clinical	[121]
Curcumin	Docetaxel	Metastatic castration-resistant prostate cancer	No therapeutic benefit	Clinical	[130]
Curcumin	Aloe Vera gel	Oral Submucous Fibrosis	Anti-inflammatory	Clinical	[118]
Curcumin	Piperine	COVID-19, ischemic stroke	Increased bioavailability and an energy booster	Preclinical and Clinical	[108,109,110,111]
Curcumin	Alendronate	Osteoporosis	Anti-inflammatory	Clinical	[132]
Curcumin	Dexamethasone and hyaluronidase	Oral Submucous Fibrosis	Anti-inflammatory	Clinical	[119]
Curcumin and curcuminoids, entrapped in a patented delivery system (LipiSperse^®^)	Ferrous sulphate	Inflammation	Anti-inflammatory effects	Clinical	[123,125]
Curcumin and curcuminoid	Blue light, Sodium dodecyl sulphate	Oral disinfectant	Reduction in salivary microorganisms	Clinical	[115,116,117]
Curcumin	Resveratrol	Malnutrition	Increase in bone and muscle mass, reduction in fat	Clinical	[134]
Curcumin and desmethoxycurcumin and bisdemethoxycurcumin	folinic acid-5-fluorouracil-oxaliplatin chemotherapy	Metastatic Colorectal Cancer	Improves the quality of life of metastatic colorectal cancer patients	Clinical	[128,129]
Curcuminoids and Turmeric oil	Diclofenac	Knee osteoarthritis	Tolerable and analgesic	Clinical	[135]
Curcuminoids extract	Hydrolysed collagen, and green tea extract	Osteoarthritis	Modulates key catabolic, inflammatory, and angiogenesis factors associated with osteoarthritis progression.	Clinical	[131]
Nanocurcumin	Omega-3 fatty acids	Episodic migraine	Reduce serum VCAM level	Clinical	[136]
Turmeric Phytosome^®^ (equivalent to 10 mg of curcumin)	*Boswellia serrata* (BSE) gum resin	Oxidative stress and inflammation	Anti-inflammatory and antioxidant effects	Clinical	[122]
Turmeric volatile oil	Boswellic acid extract from Indian frankincense root	Osteoarthritis	Analgesic	Clinical	[133]

## 6. Conclusions

In conclusion, the synthesis of evidence presented in this review highlights the remarkable potential of nanoencapsulation strategies to address the challenges associated with curcumin therapeutics. By enhancing solubility, bioavailability, and therapeutic efficacy, nanodelivery systems offer a promising avenue for overcoming the limitations of conventional curcumin formulations. Moreover, the synergistic effects observed through the combination of curcumin with other bioactive compounds shows the value of combinatorial approaches in augmenting therapeutic outcomes across various disorders. While significant progress has been made in elucidating the pharmacokinetic profile and therapeutic applications of curcumin, further research is warranted to optimize nanoencapsulation techniques and explore novel drug combinations. Future studies should focus on refining nanodelivery systems to maximize drug loading capacity, improve targeted delivery, and minimize potential toxicity. Additionally, investigating the mechanisms underlying the synergistic interactions between curcumin and other compounds can provide valuable insights into designing tailored therapeutic interventions. Overall, this review shows the importance of continued exploration and innovation in curcumin therapeutics, with nanoencapsulation and combinatorial approaches holding great promise for advancing the field and ultimately improving health outcomes for individuals worldwide.

## Data Availability

All data are provided in the manuscript.
